# Housing for Female Factory Workers: The Association between Renting Accommodation and Satisfaction with Income and Living Conditions

**DOI:** 10.3934/publichealth.2016.4.837

**Published:** 2016-10-10

**Authors:** Quynh Thuy Nguyen, Thuy Thi Thu Tran, Chinh Thi Thuy Phan, Tuan Cong Pham

**Affiliations:** 1 Department of Occupational Health and Safety, Hanoi School of Public Health, Hanoi, Vietnam; 2 National Institute of Occupational and Environmental Health, Vietnam; 3 Centre for Environment and Population Health, Griffith University, Australia

**Keywords:** house renting, income satisfaction, living conditions, female workers, light manufacturing industry, Vietnam

## Abstract

**Background:**

Vietnam has experienced a strong wave of migrants to urban and industrialized areas. This is a challenge for both local and national governments, which need to address the problems of the poor and socially marginalized, including providing housing for rural-to-urban migrants. Poor housing and the economic burden of house renting are increasingly recognized as determinants of both physical and mental health.

**Objectives:**

This paper examined the association between renting accommodation and income satisfaction and living conditions of female workers in light manufacturing industries in Vietnam.

**Methods:**

A cross-sectional study was implemented with quantitative survey of 2,818 female workers in 10 light manufacturing factories in 3 industrial zones by a self-administered questionnaire.

**Results:**

Over 38% of female workers had to rent accommodation. The average expense for accommodation, water and electricity accounted for 30.1% of renters' income, which is 7.2% (CI 95%, 5.3–9.3%) higher than for non-renters. A higher proportion of renters than non-renters considered their income was unstable and insufficient for living costs. In addition, only 7.2% of renters reported that their living conditions were suitable, notably lower than non-renters (22.4%).

**Conclusion:**

The study showed the economic burden of renting accommodation on workers' income satisfaction and living conditions. The findings have implications for an adequate housing access strategy for workers including the integration of housing development in the planning and development of industrial zones and factories.

## 1. Introduction

Like many developing countries, Vietnam has undergone a substantial socioeconomic transition. In 30 years of “Doi Moi”, the economic reforms and international economic integration have altered Vietnam from one of the world's lowest income countries to a lower-middle-income country [1],[2]. However, several studies indicate that the economic reforms have widened economic disparities across Vietnam's regions [1]. Uneven distribution of wealth and opportunity and lower cost of travel has led to growing numbers of internal migrant workers [2]–[4]. In addition, the development of manufacturing industries and production systems in developing and emerging economies has added new waves of internal migrants [5],[6]. In 2010, Vietnam had 260 industrial parks, nearly four times more than in 2001 [7],[8]. The concentrations of industrial zones and large cities are always a strong attraction for wage workers and low-skilled workers [9]. In 2009, there were 6.7 million internal migrants, accounting for approximately 8.6% of the total population [10],[11]. Population census data suggest that the number of migrants will reach more than 10 million people in 2019; of these, six million will be inter-provincial migrants, representing 6.4% of the total population [10],[11].

A significant proportion of internal migrants in Vietnam are economic migrants, mostly to work for growing light industries and service sectors in urban areas [12]. Some light industries, such as manufacturing of electronic devices, leather products, clothes, and food products, have a very high proportion of internal workers [13]. With such a strong wave of migration to urban areas, a challenge for both local and national governments is how to address the problems of the poor and socially marginalized, including rural-to-urban migrants [1]. Such migration will cause employment, health, housing and social problems in the urban and suburban areas [14]. Moreover, government policies and laws and agencies have not paid enough attention to protecting the right of migrants to access basic social services [11],[15]. One particular policy which adversely impacts internal migrants and their rights is the “hộ khẩu”, or household registration system, which records and restricts changes in people's residency by classifying households into different categories with differential entitlements, such as the ability to access basic social services [12]. National policies have long been established on locality-based schemes that depend on household registration, including housing policy and development planning [16].

Providing adequate and affordable housing for rural-urban migrants is one of the policy challenges neglected in urban development in Vietnam [12],[17]. A report of the International Institute for Environment and Development showed that unlike migrant workers in China, who mostly live in dormitories provided by their employers, migrant workers in Vietnam often live in accommodations which are privately owned and developed by local owners without any legal standard required [18]. Instead of large-scale “slums” in Vietnam's cities (though small slums exist), there are many very small and poorly-equipped living spaces, rented to poor migrants [17]. Those living spaces are often small rooms, surrounded by pollution with bad sanitation, muddy access roads, poor water quality and unreliable electricity. Several qualitative studies in Vietnam have shown that migrant workers who lived in rented accommodations express their dissatisfaction with their living condition due to the low quality of the rented rooms. Migrant workers report “uncomfortable and unsafe living conditions”, with two to four people a room and the thin walls and “fibro” (fibrous cement sheet) roof mean the small room (around just 10 m^2^) is very hot in summer [17]. Migrant women are more disadvantaged than men when living conditions are poor, finding it more uncomfortable than men, for example, to use shared toilets and bathrooms [17].

Apart from the poor living condition, the rental expense has been described as a major economic burden for internal migrant workers. Siu and Chan argued that many strikes in the manufacturing factories in Vietnam industrial zones were a consequence of dissatisfaction of wages and social benefit [19]. They also noted that the Vietnamese state's macroeconomic policy and inability to control inflation are partly responsible for the deteriorating conditions [19]. In addition, income dissatisfaction has been a significant factor contributing to the poor mental health of migrant workers [20].

With the exception of limited news media and qualitative studies, very little information is available regarding housing for migrant workers in hastily urbanized areas around industrial zones in Vietnam. This study reports the findings from a survey of female workers in industrial zones in Hung Yen province, Da Nang city and Dong Nai province in Vietnam in 2014. The study aims to describe the housing status of female workers in industrial zones and to examine the association between renting accommodation and self-reported income and living conditions satisfaction of female workers in light manufacturing industries in Vietnam.

## 2. Methodology

### 2.1. Sampling and Data Collection

The study was conducted in 10 factories, each with 400 or more female workers, in three industrial zones in three cities/provinces in 2014, representing the 3 main economic regions of Vietnam: 4 factories in Hung Yen province (Northern region), 3 factories in Da Nang city (Central region) and 3 factories in Dong Nai province (Southern region). These cities/provinces have a large number of industrial zones and high migration rates [10]. The factories were selected from the light industries that have the highest rate of female workers, namely garment, footwear, food processing and electrical device manufacturing industries. According to the Vietnam General Statistics Office, female workers account for 75% of the total working population in the garment industry and electrical device manufacturing industries, 85% in shoe manufacturing and 85% in the seafood processing industry [21],[22].

A quantitative survey was conducted using a self-administered structured questionnaire. The inclusion criteria for participants were female workers who had a work contract and had worked in the production lines of those factories for more than 3 months. The three-month duration of working enables the study to capture the impacts of working and living conditions.

The sample size for each province was calculated to estimate for a proportion and with an absolute precision, the following parameters were considered for calculating the sample size: anticipated prevalence of health problem among female workers as 35%, and absolute precision of 4.5%, an estimated non-response rate of 10%, and a design effect of 2. In each factory, a sample of 320 subjects was selected by the method of systematic random sampling from the list of the company's female workers. The sampling intervals were calculated using the companies' number of female workers and the company's sample size. A total of 2,818 research subjects were surveyed, including 940 from Hung Yen, 955 from Da Nang and 923 from Dong Nai.

### 2.2. Measurements

The data were collected via an instrument developed specifically for the purposes of the study. It was constructed in several sections: the workers' social and demographic characteristics, income and living conditions and housing status. Housing status was measured by asking the question “Where do you live?” with 7 possible responses including 3 responses for non-renters as “in your own house”, “in your parent's house” and “in your relative's house (without rental pay)”; three responses for renters as “renting accommodation and living alone”, “renting accommodation, living with family” and “renting accommodation, living with friends”; and an “other” category. Income satisfaction was measured using the question “How does your income guarantee for a decent living expense for your family?” with three possible responses: “adequate”, “barely adequate”, and “inadequate”. Living condition satisfaction was measured by the question “How do you assess your living condition?” with three possible answers: “desirable”, “acceptable”, and “unacceptable”. The questionnaire was pre-tested and then revised to suit the context of female workers in industrial zones.

### 2.3. Data Management and Analysis

The analysis of quantitative data from the survey included descriptive statistical analysis (such as frequencies, percentage and cross-tabulation). The potential relationships between house renting and income and living condition status were tested by Chi-Square tests. All the analysis was carried out using SPSS software version 20 and at 95% confidence interval.

### 2.4. Ethics Approvals

This study obtained ethics clearance approval from the Institutional Review Boards of Hanoi School of Public Health (IRB reference number 171/2013/YTCC-HD3). The participation in the study was completely voluntary and the participants were informed that they could withdraw at any time. The participants were offered a small incentive for participating the survey. Standard consent forms for participation were obtained from all the eligible respondents.

## 3. Results

### 3.1. Participants

Table 1 presents the distribution of respondents by main sociodemographic characteristics of the sample. The mean age of female workers was approximately 30 years of age (±8.14). More than 97% of women were in reproductive age (18–49 years old); only under 1% of female workers were under 18 years old and 1.7% were over 49 years old. Notably, more than three in four female workers were in the age group of 18–35 years of age. Two-thirds of the women (77.8%) had a secondary education. Only 17.6% of the women had vocational, graduate or higher education. Of the 2818 women involved in the research, 1898 female workers (67.4%) were married. Among married women workers, most (87.6 %) of them already had 1–2 children.

**Table 1.** Socio-demographic characteristics of sample.

Characteristics	n	%
**Average age (years)**	**29.86; SD: 8.14**	
**Age groups (years)**		
<18	27	1.0
18–35	2,125	75.4
>35–49	617	21.9
>49	49	1.7
**Education level**		
Primary	132	4.7
Lower secondary	843	29.9
Higher secondary	1,349	47.9
Vocational training	272	9.7
College and higher	222	7.9
**Marital status**		
Single	920	32.6
Married	1,818	64.5
Divorced	80	2.8
**Number of children**		
0	1,115	39.6
1–2	1,592	56.5
>2	111	3.9

Among the 2,818 female workers in the sample, 83.7% had an income higher than 3 million Vietnam dong/month (Around 150 USD) and. the rest (14.3%) earned between 1.8 and 3 million Vietnam dong per month. Income distribution of female workers varies between industries. The proportion of female workers earning more than 3 million/month was highest in the footwear industry (92.4%), followed by the electrical devices manufacturing industry (89.8%) and the garment industry (80.7 %). This proportion is lowest in the seafood processing industry (63.7%).

### 3.2. Housing and Living Condition

Table 2 shows that over 38% of female workers rented accommodations. Among renters, the majority shared the accommodation with family (39%) or friends (33.2%). Only 27.7% of renters lived alone. In addition, only 2 female workers, accounting for only 0.1% of the sample, reported that they lived in the dormitories provided by the company.

**Table 2.** Housing status of workers.

Housing status	n	%
**Non-renters**	**1,736**	**61.6%**
**House owner**	1,145	40.6%
**Living with parents**	546	19.4%
**Living with relatives**	45	1.6%
**Renters**	**1,076**	**38.1%**
**Renting, living alone**	300	10.6%
**Renting, living with family**	418	14.9%
**Renting, living with friends**	358	12.6%
**Dormitories**	**2**	**0.1%**
**Missing**	**4**	**0.2%**

There are statistically significant differences in the housing situation of women workers in the three jurisdictions (Chi-Square = 489; *p* < 0.001). The proportion of the female workers who lived in rented accommodations is highest in Hung Yen (60.1%), followed by Dong Nai to (43.2%) and lowest in Da Nang (11.5%).

As noted earlier, 60.1% of female workers in industrial zones in Hung Yen were renters. The largest number of the female factory workers in Hung Yen rented with friends or family (35.7%) or rented alone (24.4%). Meanwhile in Da Nang, the majority of the female workers lived in their own homes (65%) or stayed with the parents (21.5%). Only 11.5% of Da Nang women lived in rented accommodations. In Dong Nai, although the proportion of women owning their own home is relatively high (36.2%), 43.2% of women have to rent accommodations, mostly living with family (23.7%) or friends (13.4%).

Among renters, 29.5% of female workers lived in accommodations without separate toilets, 18.7 percentage points higher (CI: 15.8–21.5%) than the proportion among non-renters (10.8%).

**Table 3.** Self-rated living condition by accommodation renting status (Chi-Square = 184.5; *p* < 0.001).

		Self-rated living condition			Total
		Desirable	Acceptable	Unacceptable	
Non-renters	n	388	1,329	19	1,736
	%	22.4%	76.6%	1.1%	100%
Renters	n	77	912	87	1,076
	%	7.2%	84.8%	8.1%	100%
Total	n	465	2,241	106	2,812
	%	16.5%	79.7%	3.8%	100%

Table 3 shows that only 7.2% of renters reported that their living conditions were suitable and comfortable, notably lower than non-renters (22.4%). In addition, 8.1% of renters reported that their living places were not suitable or comfortable, statistically higher than the proportion among non-renters (1.1%).

### 3.3. Income Satisfaction

The majority of women (82.8%) reported that their income is “adequate” or “barely adequate” to cover basic family expenses. Few women workers (2.8%) said that their own income could provide more than basic living for their family, while 14.4% of the women said that their current income is not enough to cover basic family expenses. A higher proportion of renters considered their income was insufficient for living costs than non-renters. Over 28% of female workers in industrial zones who had to rent accommodation claimed that their income was insufficient for living expenses, significantly higher than the proportion among non-renters (18.5%).

**Table 4.** Self-rated income satisfaction by accommodation renting status (Chi-Square = 36.4; *p* < 0.001).

		Self-rated income satisfaction			Total
		Adequate	Barely adequate	Inadequate	
Non-renters	n	51	1,364	321	1,736
	%	2.9%	78.6%	18.5%	100%
Renters	n	29	745	302	1,076
	%	2.7%	69.2%	28.1%	100%
Total	n	80	2,109	623	2,812
	%	2.8%	75.0%	22.2%	100%

In addition, the expense for accommodation was a significant burden for female workers in industrial zones. The average expense for accommodation, water and electricity accounted for 30.1% of renters' income, which is 7.2 percentage points higher (CI 95%: 5.3–9.3%) than for non-renters.

## 4. Discussion

The majority of women workers in the industrial zone were young. Most of the female workers in the sample were from 18 to 35 with an average age of 30 years. Other studies in Vietnam also showed similar results. Workers aged 18 to 30 years old accounted for 36.4% of the total; especially in the foreign investment enterprises, workers under 25 years old accounted for 43.4%, and those 26–35 years of age accounted for 34.7% [23]. Other studies in China have shown that workers in the industrial zones are mainly young women of reproductive age, with 73.3% under the age of 30 [24].

This study has identified that large proportion of workers in industrial zones had to live in rented accommodation. In addition, the quality of rented accommodations for workers in industrial zones is very poor both in terms of sanitation and comfort. The similar result could be found in other reports. Loi reported that the majority of migrant workers in industrial zones shared a renting house with limited facilities, either semi-permanent, or wood frame, or simple structured houses, which have poor sanitation and sharing toilet (16% and 36% of Northeast and in the Southeast Industrial Zone, respectively) [25]. This finding also reflects the results of research on poor housing condition of the urban poor reported in Urban Poverty in Vietnam [17],[26]. This situation is similar to other fast industrializing and urbanizing societies such as China [27]–[29].

In addition, very few female workers reported that they lived in dormitories or accommodations that are developed by the government or factories. Some studies from China have shown that, despite some disadvantages of living in dormitories provided by factory owners, factory dormitories are an affordable and adequate source of housing for poor migrant workers [30]. Therefore, housing development should be considered as a part of industrial zone development.

Female workers who lived in rented accommodations expressed their dissatisfaction with their living conditions. Poor urban living conditions and dissatisfaction with income are sources of stress among migrant workers. Other research has shown that rural-urban migrant workers in Hanoi, Vietnam described their living condition as “crowded, worrying, dissatisfying and disgusting” and perceived their poor living condition as a source of ill-health [31]. In another qualitative study, female workers in the industrial areas described their rented accommodations as a small room with an area of 12–15 m^2^, and they often share this room with friends and family members. In addition, most of the living activities were done in the single room accommodation including cooking, entertaining and sleeping [32].

Previous research papers in Vietnam have also shown that sanitation and safe water were also significant concerns of female workers who lived in rented accommodations in the sub-urban areas around industrial zones. Similarly, others reported that the use of public toilets could potentially cause unhygienic risks and was uncomfortable for female migrant workers [17],[32]. Female migrant workers expressed the concern about reproductive tract infections and infectious diseases from using public toilets. Female migrant workers also faced issues in terms of perceived security when using public toilets and bathroom facilities [17],[32].

Spending for renting also posed a substantial economic burden for female workers. Renters had to spend more on housing and utility bills than non-renters. As a consequence, renters reported a lower level of income satisfaction than non-renters. To date, income dissatisfaction has been identified as a source of social unrest and mental health problems by some studies in Vietnam. As noted earlier, many strikes in industrial zones in Vietnam are due to the conflict over wages and income because workers claim that their income is unable to cover their basic living expense [33]. Other research has shown the burden of inflation rate on rental prices in industrial zones as it affected the workers' ability to provide basic living needs for their family [19]. Income dissatisfaction is also associated with poor mental health in migrants in China [20]. Other studies among internal migrants in Brazil [34] and Thailand [35] also report an association between the better economic opportunities due to migration and a positive mental health status. Obviously, the neglect of housing policy of the authority in industrial zones and factories has increased the vulnerability of female workers [36],[37].

The findings of this study should be interpreted in the light of its strengths and limitations. The study was confined to data in enterprises with more than 400 workers. The results would be more representative if the sample included of women working in small and medium-sized businesses and freelance workers in and the sub-urban areas surround the industrial zones who might experience different aspects of vulnerability [38]. Moreover, the study results reflect only the current status of income and living condition satisfaction from the perspective of female workers. This is the first large size study conducted among female workers in industrial zones in Vietnam, therefore the research had to build data collection toolkits. In addition, the involvement of medical staff and safety officers of the company and local health care officers potentially created response bias. To limit this problem, the study used a self-administered structured questionnaire to collect sensitive information such as quality of life and living conditions.

## 5. Conclusion

Large proportion of workers in industrial zones had to live in rented accommodations. In addition, a large number of female workers in three industrial zones in Vietnam have expressed their dissatisfaction with the poor living conditions in their rented accommodations. The study also showed the significant economic burden of house renting on workers' income satisfaction. The findings have implications for an adequate and affordable housing access strategy for workers including the integration of housing development in the planning and development of industrial zones and factories.
